# Traditional health practitioners’ training needs on biomedical knowledge and skills in a South African township

**DOI:** 10.4102/phcfm.v15i1.3923

**Published:** 2023-11-01

**Authors:** Mabitja Moeta, Maurine R. Musie, Raikane J. Seretlo, Maikeleng Ledimo, Melitah M. Rasweswe, Eugene Makhavhu, Fhumulani M. Mulaudzi

**Affiliations:** 1 Department of Nursing Science, Faculty of Health Sciences, University of Pretoria, Pretoria, South Africa; 2 Department of Nursing Sciences, Faculty of Healthcare Sciences, University of Limpopo, Polokwane, South Africa; 3 Department of Nursing Science, School of Healthcare Sciences, Sefako Makgatho Health Sciences University, Pretoria, South Africa

**Keywords:** traditional health practitioner, biomedical health practitioners, training needs, biomedical system, knowledge and skills

## Abstract

**Background:**

Traditional health practitioners (THPs) play an important role in communities by providing necessary health services for a variety of health problems. Possessing complementary biomedical knowledge and skills is vital in saving lives of patients. However, less is known about biomedical knowledge and skills among THPs.

**Aim:**

This study aimed to explore and describe the training needs of THPs on biomedical knowledge and skills in urban townships in South Africa.

**Setting:**

The study was conducted in a township in the City of Tshwane Metropolitan Municipality of Gauteng province, South Africa.

**Methods:**

A qualitative, explorative, descriptive design with 18 THPs was employed through snowballing sampling. Data were collected through a lekgotla group discussion and thematic content analysis undertaken.

**Results:**

Themes that emanated include knowledge of the basic physiological functioning of the human body; biomedical knowledge and skills required for the assessment of patients; managing emergency health conditions and understanding diagnostic concepts used in traditional health practice versus biomedical systems.

**Conclusion:**

Traditional health practitioners have demonstrated interest in being trained on certain skills used within the biomedical system to care for patients. Performing the necessary first-aid skills by THPs will assist patients in the communities while waiting for emergency services or referrals. Provision of training programmes for THPs on first aid interventions during emergencies is therefore recommended.

**Contribution:**

The study revealed that capacitating THPs with biomedical knowledge and skills can improve their ability to promote healthy living and prevent health problems in communities where access to resources is limited.

## Introduction

Traditional medicine offers solutions to people’s health needs and complements the primary level of care in most African communities.^1,2^ In the communities, healthcare services provided by traditional health practitioners (THPs) are often tailored to meet the health needs of the served population. Such services have been provided before the advent of modern Western medicine in Africa for preventing illness, promoting health, treating minor and chronic ailments as well as rehabilitative services.^3^ However, there are instances where the level of knowledge and skill of THPs has been viewed by biomedical health practitioners (BHPs) as impediments to the provision of urgent health interventions and referrals. There are reported cases, where THPs have been blamed for delayed care of patients who turn up at health facilities with complications that may have been avoided, had they referred patients earlier or if they possessed knowledge and skills to conduct a baseline biomedical assessment.^4,5,6^ Traditional health practitioners have their own methods for diagnosing illnesses, including divination and bone throwing for ancestor guidance during consultations and prayer, as well as their own methods for treating patients, including the distribution of herbs and rituals.^7^ Traditional health practices have been used in combination with biomedical knowledge and skills with either satisfactory or opposed outcomes.^8^ Therefore, possessing biomedical knowledge and skills by THPs may help them in early identification and referral of patients.

Having knowledge and skills in biomedical procedures would mean that THPs can obtain information on vital signs or parameters of the patient’s health condition, such as temperature, pulse, respiration and hydration status and detect any signs of a patient in shock.^9^ These vital signs are important in diagnosing serious life-threatening conditions and for health providers to prioritise patients in need of urgent or prompt care. In Africa, THPs often attend to patients with serious health conditions that require immediate health interventions using different herbs and medicines that are now used to treat patients with a variety of illnesses, such as patients with malaria,^10^ burns, febrile illnesses,^11^ poisoning,^12^ HIV-related opportunistic infections, acute or chronic bleeding and stroke.^13^ For some patients, the ability of the THPs to diagnose the seriousness of the presenting issue and offering life-saving measures could spell the difference between life and death, especially because THPs are mostly the first point of care for many African patients before they go to biomedical health services.^1,5,14^

The role and importance of THPs in providing primary health care services to poor communities, where there is a shortage of resources and access to facilities is costly, have been reported.^3,15^ In contributing to timely health interventions, THPs must be able to detect certain abnormalities that may constitute a medical emergency and initiate life-saving interventions on their patients. Competency in providing such services is vital in contributing to reduced morbidity and mortality among patients who consult such THPs.

The contribution of THPs in managing several conditions, such as malaria, anaemia, cholera, dehydration and fever, by using local herbs and referring patients they could not manage in time to the hospital has been reported.^16^ Nonetheless, in Sierra Leone, THPs are compelled to send very sick patients to hospitals as it is an Ebola-stricken country and therefore results in many fatalities at their hands when they attempt to treat such patients.^17^ Patients who are very sick in Sierra Leone must be treated at the hospital and be given clearance documents before being attended to by THPs because of previous cases where THPs are reported to have died attempting to treat diseases such as Ebola.^18^ Yet, there are reported cases where THPs have been trained on triage and diagnosis of fatal conditions such as malaria and Ebola.^17^ Upon arrival to the practice by patients, the THPs who are knowledgeable and skilled should be able to detect immediate danger signs in patients such as responsiveness, skin colour and abnormalities in vital signs such as fever, abnormal breathing and pulse. The ability of the attending THP to triage patients based on their health condition and noticing emergencies becomes paramount to avert complications associated with delayed patient care.

Contrary to biomedicine, in traditional medicine, the training of THPs focuses on diagnosing the causes of illness from a variety of sources such as evil spirits or curses and the displeasing of ancestors.^19^ In some instances, the belief in the aetiology of illness may be an impediment where the patient’s health condition may require immediate resuscitation, and the physical needs of the patient supersede the spiritual intervention. Undoubtedly, THPs play an important role in line with primary health care re-engineering, where they continue to offer some relief to the health system in clinics and district hospitals.^20^ However, they do not have formal recognition as healthcare professionals as yet in South Africa. Although THPs receive training during their initiation, their level of training has been questioned on whether it can be compared to the training of practitioners from Western medicine.^21^ Within traditional healing, certified training programmes rarely exist, as it is believed that the training is guided by ancestral directives.^22^ This poses challenges when efforts are made to locate the role THPs can play within the mainstream health system. Exploring the training needs of THPs provides a clearer picture of areas that need to be targeted to support their efforts and contribution to the mainstream health system.

## Aim

This study aimed to explore and describe the training needs of THPs on biomedical knowledge and skills in urban townships in South Africa.

## Research design and methods

### Research design

The study adopted a qualitative, exploratory and descriptive research design using a *lekgotla* discussion to engage the participants. A qualitative research design was used because little is known about the training needs of THPs and to allow the participants and researchers to engage so that their authentic views can be heard and shared.^23^ *Lekgotla*, as a qualitative method of collecting data, involves indigenous leaders facilitating consensus by collectively engaging in robust discussions with the participants thereby improving the rigour of the data collection method.^24^ Therefore, *lekgotla* was seen as an appropriate data collection method that the THPs are familiar with because they are also community indigenous knowledge and leaders.

### Study setting

The study was conducted in the City of Tshwane Metropolitan Municipality of Gauteng Province, South Africa. The province of Gauteng has rural, urban and semi-urban areas, with diverse cultural and traditional beliefs and practices. There are approximately 11 district municipalities in Gauteng Province, including the city of Tshwane. Most of the THPs are in rural areas and urban townships, where the dominant residents are black African people. Residents living in these townships are known to utilise the services of both the traditional health and Western health systems. Gaining access to an indigenous research setting requires that researchers be aware of potential barriers or impediments such as principles for establishing rapport, negotiation and compromise approaches, their perceptions about traditional health as well as expected dress code.^25^ In this study, to gain access to participants, researchers approached a local THP who collaborates with the University of Pretoria to ask about other THPs practising in the study setting. The purpose was also to understand the research settings and expectations when visiting other THPs as well as acceptable approaches for communication and information sharing within the indigenous research setting. Consequently, researchers obtained rich data from the lead THP, which facilitated the building of mutual trust and the establishment of common ground and understanding within the settings. Researchers were made aware by the lead THP of the cultural practices that are important for building trust and fostering open communication such as salutations, language use and dress code expectations during interviews when interacting with THPs.^23^

### Study population and sampling method

The study participants comprised both male and female THPs. It was previously estimated that there were between 150 000 and 200 000 THPs in South Africa.^26^ There is limited information on the number of THPs in Gauteng Province and the City of Tshwane. Snowballing sampling method was therefore used to sample the participants who were known by the leading THP, who was asked to refer researchers to other private practising THPs in the townships for recruitment. After receiving the names and contact details of prospective participants, the researchers collated a list of the THPs and arranged a common date for all participants where a meeting was held with the potential participants to share with them the purpose of the study and identify those who were willing to participate in the study. Both male and female qualified THPs and student initiates from the city of Tshwane municipality were included. The participating THPs would have been in practice for a year and above to be included. Traditional health practitioners who did not give consent to participate or those outside the setting of the study were excluded. In the initial meeting, after consent to participate was obtained, demographic data were collected regarding the participant’s gender, age, years in practice, category of practice, number of patients seen in a month, treatment methods and common health problems treated. The date for *lekgotla* was then decided and eighteen participants consented to be part of the *lekgotla* discussion.

### Data collection

The study adopted a *lekgotla* discussion as a method of data collection to draw on the debates and dialogue where participants in a collective group engage in an open discussion based on the issues raised.^27^ *Lekgotla* discussion is a form of participatory approach to discuss pertinent issues, in which the participants are involved in identifying opportunities that are helpful to address a social change.^28^ Historically, traditional leaders have used this method to address community members with any issues that affected the community.^24^ Other researchers used the *lekgotla* discussion successfully for health research involving indigenous people as they find it culturally appropriate and in line with the participants’ ways of knowing.^29^ The *lekgotla* method is a common method of problem-solving and engagement used among Africans in seeking consensus by adopting participatory processes.^30^ The *lekgotla* discussion allows the participation of the whole cohorts or representative groups of participants without looking at the numbers.^23^ Hence, the proceedings of the *lekgotla* may begin once every expected participant has arrived.^24^ Despite the number of participants, the debates and dialogues in a *lekgotla* discussion are effective to generate large numbers of creative new ideas and information that contribute to knowledge. In this study, *lekgotla* discussion was used as a means of cultural respect and promotion of collaborative partnership with THPs.

The meeting was held in the yard of the leading THP. The engagements were conducted in English as most participants are conversant in the language. All participants, including researchers, were seated in a circle to promote equity.^30^ The discussion was facilitated by the lead author and the leading THP who was trained by the lead author on how to facilitate. The leading THP did not participate in the discussion. To commence the *lekgotla* discussion, participants were first asked if ever they experience challenges when assessing health needs of their patients. They were also probed depending on the provided answers. This led to the conclusion that the participating THPs have training needs on biomedical knowledge and skills. Therefore, they were asked about the training needs that this paper is reporting. The central question of this study was: *What are your training needs as THPs on biomedical knowledge and skills?* The question was asked so that the *lekgotla* discussion should remain focussed on the THPs’ training needs. During data collection, the leading THP, who is also conversant in English and traditional health language, assisted in translating the information that was being shared by participants to provide context and clarity. Traditional Health Practitioners were also encouraged to express themselves in their vernacular so that meaning of their input is not distorted The concept of biomedicine was subsequently explained to guide the responses of THPs and clarify any unclear explanations. Involving the leading THP as a co-facilitator ensured that the discussion shifted towards open dialogue and ownership of the discussion was transferred to participants. Participants took turns by raising their hands and allowing them to be recognised by the facilitator prior to sharing training needs. The lead author also ensured that participants who were not raising their hands were asked to share their ideas as during *lekgotla* every member is supposed to deliberate issues under discussion to the best of their understanding of the topic.^30^

In between the discussions, the facilitators (researchers and the leading THP) directed and monitored the discussion process through probing, so that the ideas raised were understood within the context of the discussion. As the participants were sharing their training needs, all the researchers (individually) collected field notes to further understand the training needs identified by the participants. The lead author ensured that all the participants contributed to the discussion and conclude if no more new data were forthcoming out of the engagement. Once all the participants had shared their ideas and there was no repetition of the same information from the probing, the facilitator summarised the inputs from the discussion. The participants were further engaged in debates based on the summary of the ideas shared by the facilitator and each participant was asked to comment by adding new information from the summary. The process was repeated until all the participants agreed that there was no more new information to be discussed. This was to ensure that there was data saturation. The *lekgotla* discussion was audio recorded as permitted by participants. The debates and dialogues assisted participants to agree on their identified needs without undermining the knowledge and roles they play in their practices.

### Data analysis

The data were analysed using thematic content analysis. Thematic analysis approach was used, as it enabled the researcher to explore the meaning that participants attached to their lived experiences and how they constructed their social world through meaning-making.^31^ Themes were captured to illustrate what is important in relation to the research question and appeared more than once in the collected data. A rich detailed description of themes was provided confirming that data saturation was achieved.^32^ The themes were extracted after reading and re-reading, analysing and interpreting the data. Similar ideas were grouped together, by identifying frequent patterns in the codes.^31^ To find the meaning of the experienced phenomenon, meaningful statements from the themes and subthemes were formulated.

### Ethical considerations

Permission to conduct the study was granted by the Faculty of Health Sciences Human Research Ethics Committee at the University of Pretoria (protocol 192/2020). All methods were carried out in accordance with the relevant guidelines and regulations. Written informed consent was obtained from all the participants. Participants were given information on the nature of the study, its intended purpose and data collection methods. In the consent form, participants were advised to indicate if there is any information that they shared, which needs to be kept strictly confidential. Permission was also sought for publishing information in an article format, and there was no objection from all the participants.

## Results

### Demographic characteristics of the participants

A total of 18 THPs participated in the study. In terms of the age of participants, the oldest THP was 63 years old while the youngest was 34 years old with 40.3 as the average age. Gender-wise, 15 (83%) THPs were females and 3 (17%) were males. Regarding the category of practice, 3 (16.6%) were identified as herbalists, 12 (66.6%) as diviners, 2 (11.1%) were students undergoing initiation and 1 (5.5%) was a general practitioner.

Regarding years in the practice category, the highest number of years was 30, while the least was 1, including student initiations. The average for the years of experience, excluding two students, was 10 years, while cumulatively all THPs provided consultation to 144 different patients monthly. Results also indicated that THPs use consultation methods such as appointments made by patients, phone calls, self-referrals, referrals by other THPs, appointments and walk-ins by patients. Regarding the commonly used methods of treatment, THPs reported that they use herbs, animal products, prayer and *iSiwasho* (healing water). Counseling of patients by THPs was also added as a complementary service. Table 1 shows the demographic profile of the participants.

**TABLE 1:** Demographic data of participants.

Participant code	Age	Gender	Category of practice	Years in practice	Number of patients provided primary care services in the past month	Consultation method	Common treatment methods used
THP1	36	F	Diviner	2	8	Appointment, call	Herbs
THP2	45	M	Diviner	6	12	Self-referral	Animal products, herbs
THP3	50	F	General	16	13	Call, self-referral	Herbs, healing water, prayer
THP4	45	F	Herbalist	8	29	Appointment, call	Herbs
THP5	38	F	Diviner	4	7	Appointment	*iSiwasho*, prayer
THP6	40	F	Herbalist	2	3	Appointment	Herbs
THP7	33	F	Diviner	5	12	Self-referral	Healing water, *iSiwasho*, prayer
THP8	63	M	Diviner	24	5	Appointment	Herbs, animal parts
THP9	57	F	Student (initiate)	1	Under supervision *uGobela* (trainer)	Self-referral	Herbs
THP10	52	F	Student (initiate)	1	Under supervision *uGobela*	Self-referral	*iSiwasho*
THP11	34	F	Diviner and/or social worker	6	4	Call and/or referral	Healing water and/or counselling
THP12	47	F	Diviner	11	10	Referral, call	Herbs, healing water
THP13	59	F	Diviner and/or Cosmologist	30	13	Call and/or self-referral	*iSiwasho*, herbs counselling
THP14	39	F	Herbalist	7	5	Appointment	Herbs, animal products
THP15	41	F	Diviner	9	5	Self-referral	Herbs
THP16	49	M	Diviner	20	20	Referral, call	Healing water, prayer counselling
THP17	55	F	Diviner	15	7	Appointment, walk in	Herbs, animal products
THP18	38	F	Diviner	8	7	Referral	Water, herbs, animal products

THP, Traditional Health Practitioner; F, female; M, male.

### Presentation of themes and sub-themes

Four themes that emanated from the *lekgotla* discussion are knowledge of the basic physiological functioning of the human body; biomedical knowledge and skills required for biomedical assessment; managing emergency health conditions and understanding terminology used in traditional health practice versus biomedical systems. The themes and subthemes discussed in this article are listed in Table 2.

**TABLE 2:** Themes and subthemes.

Theme	Subtheme
1. Knowledge of the basic physiological functioning of the human body	-
2. Biomedical knowledge and skills required for assessment of patients	2.1 Conducting a basic physical examination 2.2 Biomedical tools required to complement traditional health diagnosis
3. Managing emergency health conditions	3.1 Training in first-aid for emergencies 3.2 Referral of high-risk patients to health facilities
4. Understanding terminologies used in traditional health practice versus biomedical system	-

### Theme 1: Knowledge of basic physiological functioning of the human body

*Lekgotla*’s discussions revealed that THPs are keen on learning biomedical knowledge pertaining to the functioning of the human body and diseases. Traditional Health Practitioners reported that when they are knowledgeable about how the body functions, they can understand and consider how their interventions can influence patient outcomes.

This is what the THPs said:

‘When coming to the diseases like hypertension and diabetes mellitus we are not fully knowledgeable about how they affect the body. Most of the time, you find that the Western is saying that the person has high blood pressure and diabetes mellitus, and when we come to mix our herbs, the person ends up being okay. But we cannot explain how this happens.’ (THP6, female, 40 years)

‘I would like them to know the basics of how the body works because you find that most of them when they come, they hyperventilate, and pass out. I have to know what is wrong with them physically before I can help them spiritually.’ (THP10, female, 52 years)

Other participants revealed that in traditional health practice, there are different ways in which body functioning is assessed. Further, the medications are tailored to treat certain body parts. Hence learning about functioning from a biomedical perspective can enhance their knowledge and understanding.

Elaborating on the same, the THPs said the following:

‘The way in which we assess our patients and *le hore mere ya sebetsa, ho ya ka hore re bona motho a le jwang* [*the way we assess our patients’ condition and if the herbs work, depends on how we see the person to be*]. *Ba bang ba tla ba le weak and ba hloka mere ya ho nwa ho ba fa maatla* [*Others come being weak and need herbs to boost their energy levels*].’ (THP2, male, 42 years)

‘*Rona re rutilwe hore* [*we were taught that*], if a patient present with a problem *ya ho tsholla* [*a problem of diarrhoea*]*, bothata bo ka teng, so o hloka mere ya ho emisa letsholoho* [*the problem is in the gut, so the patient needs herbs to stop the diarrhoea*]. I am not sure [*how diarrhoea is defined*] in your medical language.’ (THP4, female, 45 years)

### Theme 2: Biomedical knowledge and skills required by Traditional Health Practitioners for assessment of patients

The second theme that emerged from this study was the biomedical knowledge and skills required by THPs to assess patients. Participants reported such knowledge and skills as important for conducting physical examinations. Additionally, THPs indicated that they require skills in using biomedical tools that help to complement their diagnosis of patients.

#### Subtheme 2.1: Conducting a basic physical examination

Traditional health practitioners reported on the biomedical knowledge and skills required to determine the extent of the problem patients present. This includes the ability to perform an assessment and knowing what to look for in the patients:

‘*Re le dingaka* [*as THPs*], we also want to know certain things from Western medicine. This is because sometimes one patient will come and he or she is weak; if I learned how to check for dangers before I waste time, then I can do something urgently to help.’ (THP1, female, 36 years)

‘You must be able to check the patient, the whole body if there are other things. When the ambulance comes, they will ask about the basic vital signs like how much is the temperature [*of the patient*] … So, we also can learn to be able to tell those vital signs.’ (THP7, female, 33 years)

Other THPs cautioned against adopting Western medicine and said:

‘In traditional medicine we have our way of doing things. We may learn certain things from Western medicine, but we cannot move away *ku imfundiso zom’khulu* [*from the teachings of the elders*].’ (THP11, female, 34 years)

‘As much as we want to learn from our colleagues, I hope we will not lose *isintu* [our way of life and/or doing things]. Western medicine has their way *yoku yenza izinto* [*of doing their things*], *nathi* we have our way. So *kumele ku be ne* balance [*there must be a balance*].’ (THP14, female, 49 years)

#### Subtheme 2.2: Biomedical tools required to complement traditional health diagnosis

Participants indicated that they need knowledge of basic medical tools such as measuring temperature, pulse and respiration rate, as well as those required for performing basic life support. Further, they indicated that having the skills for using biomedical tools increases their confidence in interventions when patients come to their practices:

‘By taking the pulse, using the thermometer and all those other things they use, we can have more options. You will first use your indigenous knowledge of taking a pulse, if you are not sure you will use the Western equipment to help you.’ (THP17, female, 55 years)

‘Yes, I agree it is also difficult for us to tell patients who are HIV [*human immunodeficiency virus*] positive that they are positive, we don’t how to say it or how to tell them. You cannot tell them, and it is not our place because we don’t have the necessary skills or machines to diagnose.’ (THP8, male, 63 years)

Another participant explained how knowledge from both biomedical and traditional health practice can be useful:

‘I need to know the issues of patients with sugar diabetes, in terms of regular blood tests. Sometimes you find that they [patients] need to continue with our traditional medicines. It ends up with a combination of both traditional and medical medicines. Sometimes when you see, they haven’t told you but when you use *ditaola* [*bones*], they [*ancestors*] advise you that this one used both medical and traditional.’ (THP12, female, 47 years)

### Theme 3: Managing emergency health conditions

Traditional health practitioners manage patients with health conditions that sometimes require them to have knowledge and skills to be able to render appropriate care as dictated by the health of the patient. Traditional health practitioners reported that they need training in first aid interventions and the referral of patients who are high risk or are severely ill during consultations.

#### Subtheme 3.1: Training first-aid for emergencies

Most participants indicated that they require knowledge of first-aid interventions so that they can manage patients who need urgent interventions for a variety of health conditions. The first-aid interventions include monitoring vital signs, performing basic life support and cardiopulmonary resuscitation (CPR).

On this issue, the participants remarked the following:

‘Let me just make an example, if any of us can fall now, do you know what is your first aid kit? What do you have in your first-aid kit? Nothing!, this person has fainted, and you are only having snuff.’ (THP8, male, 63 years)

‘If we can be taught on drip management because sometimes you can check a person and see that he or she lacks fluids in his or her body. You want to know what I can do faster to help, it is first aid, CPR to prolong life until the ambulance arrives.’ (THP6, female, 40 years)

One THP said the following regarding their training needs:

‘I mean when the patient is having fits [*convulsions*], I need to know how to calm them down [*while waiting for emergency services*].’ (THP3, female, 50 years)

#### Subtheme 3.2: Referral of high-risk patients to health facilities

It emerged that, at times, the patient’s condition may necessitate immediate referral, and THPs should be able to facilitate this referral. A variety of serious and urgent health conditions such as mental illness and high-risk pregnancy were mentioned.

One of the participants commented:

‘I hear them, and I agree that first aid and the issue of pulses are important, but we must be able to assess the patient, notice signs and symptoms, and see that the patient is having a serious illness so that we can call an ambulance or refer to the clinic.’ (THP7, female, 33 years)

THP7 and THP17 went further and said:

‘Another problem is mental illness; someone comes with this issue, and they are very uncontrollable, so you need to send them to the hospital first.’ (THP7, female 33 years)

‘A pregnant woman who is at the last stage, I treat them, and you find out that they are in labour, I don’t have the skills on checking on how far. I have to send them to the hospital so that there won’t be any complications.’ (THP17, female, 55 years)

### Theme 4: Understanding terminologies used in traditional health practice versus biomedical system

It further emerged under this theme that understanding the concepts and terms used in both biomedical and traditional health practices is important for collective understanding and facilitation of communication. In terms of terminologies and their definitions, some THPs reiterated that they do not understand biomedical terms. The difficulty to understand the terms is associated with a lack of necessary training to acquire such knowledge of complex medical terminologies:

‘I want to believe that we use different words with the same meaning. This might be confusing as in Western medicine names are used in English, like a stethoscope, we call it *lenaka* [*horn*] in our practices.’ (THP9, female, 57 years)

‘We miss each other because our language is different from your language. So, if we can be taught about the meaning of names that Western doctors use so that we speak the same language.’ (THP16, male, 49 years)

Subsequently, it was mentioned that while biomedical practitioners may translate terminologies used by THPs, they are not used within the same context. Therefore, there is a difference between biomedical definitions and those used by THPs:

‘When coming to *bolwetsi ba* [*condition of*] Sefola, they call it a sore in Western and you find that they diagnose it as sugar diabetes which ends up in amputations. Therefore, such amputations were unnecessary. Traditionally, Sefola can be treated without cutting the leg.’ (THP8, male, 63 years)

The findings under this theme demonstrate that there is immense potential for knowledge exchange and enhancing the understanding of the use of diagnostic concepts between practitioners in both systems of healthcare. Practitioners’ and patients’ common understanding of diagnostic concepts can improve communication and compliance among patients.

## Discussion

This study provides more insights into the training needs of THPs in biomedical knowledge and skills. Traditional health practitioners reported that they render health services under categories of practice such as herbalists, diviners, student initiates and general practitioners. In South Africa, the *Traditional Health Practitioners’ Act 22 of 2007* makes provision for the registration of THPs under the categories of diviners, herbalists, traditional birth attendants and traditional surgeons.^33^ Herbalists are popularly known as *Inyanga* in Isizulu, while diviners are called *Isangoma*.^3^ While the roles of THPs vary according to their calling, diviners are believed to use divination, whereas herbalists use traditional medicine or herbs to treat patients.^34^ Although there is no category of general practitioner in terms of the THPs Act, the participants who identified themselves as such indicated that they practice in all categories. Traditional health practitioners in this study also indicated that other methods of healing they use include *iSiwasho*, known as healing water used to protect a person against attack by evil spirits.^35^ Further, prayer and animal products were reported as methods used for healing. Similar healing methods were reported but included the use of traditional dance to treat ancestral spiritual possession known as *malopo*.^36^ Regardless of the category of practice, THPs consult patients with a variety of health problems where at times there are limitations in terms of the nature of the presenting problem.

Knowledge of the functioning of the human body is important for healthcare providers as it ensures the provision of safe and efficient health services.^37^ Similarly in this study, THPs highlighted knowledge on physiology as essential in improving their interactions with patients. Possessing knowledge of how the body functions, commonly known as physiology in biomedicine, provides practitioners with a clear view of the affected body systems, thereby facilitating an understanding of the seriousness of the presenting problem. Although there are numerous ways to define the body and its functioning, the language used in traditional health practice remains underdeveloped and unstructured. Hence, the need for THPs to learn about physiology within the context of biomedicine and how it relates to traditional health practice was identified. Formal training of THPs is not finalised and formalised in South Africa, but one of the key knowledge areas or competencies for THPs identified is the ability to understand the illnesses and contributing factors while considering physical, psychological and spiritual wellness.^22^

The findings from the current study indicate that THPs are willing to learn more about biomedical skills and acquire knowledge from BHPs. Based on the identified training needs, more baseline information must be collected regarding the possible barriers to capacitating THPs, especially regarding their level of education, knowledge and skills. One of the challenges identified regarding traditional healthcare practice is the lack of ‘scientific’ evidence to substantiate their knowledge and practices.^38^ Currently, there is no competency framework for THPs in South Africa, within which the competence or training needs of THPs within traditional health practice can be assessed. However, competencies of THPs such as consultation, diagnosis and holistic patient care have been identified.^22^ Yet, the competencies are developed within the context of traditional health practice and not modern medicine. This may be seen as one of the reasons why there seem to be difficulties in integrating THPs into biomedical healthcare as there is a lack of scientific backing for the methods and interventions.^39^ However, in this current study, THPs note the need to improve their knowledge and skill gap on first aid interventions to better care for their patient’s health needs and to facilitate communication with BHPs.

Within traditional health practices, tools used to diagnose diseases include the use of *ditaola* (throwing bones), water and prayer.^4,40^ The use of these diagnostic tools by THPs is often guided by ancestral directives to communicate the causes of the problems that brought the patient for consultation.^14^ Contrary to traditional health practice, in the biomedical system, instruments used include the use of technology such as stethoscopes, thermometers, data scopes for blood pressure and laboratory tests.^41^ Historically, THPs were not using any medical materials for assessment or diagnostics. However, modernisation has changed this, and the use of personal protective equipment such as gloves, aprons and goggles is reported.^42^ Interestingly in rural Nigeria, some THPs use thermometers and stethoscopes in addition to traditional methods, to diagnose patients.^39^ On the other hand, in some African countries after having undergone training, THPs have been reported to provide counselling and testing for the HIV using biomedical diagnostic tools.^42,43,^ Great potential for contributing to health services exists as proven in countries where THPs provide such crucial diagnostic information.

In the current study, basic medical interventions such as assessing vital signs, emergency childbirth, CPR and measuring patients’ blood glucose are some skills that were seen to be helpful for THPs. According to THPs, knowledge of basic medical care or commonly known as first aid may well be beneficial in ensuring and promoting safe patient care and evading unnecessary patient complications. Understanding vital signs may also be of benefit for THPs to develop diagnostic skills, particularly for patients who may be in imminent danger and need urgent medical attention. Previous studies reported that THPs and traditional birth attendants were trained as primary health care workers. This resulted in changes in health behaviours for patients after THPs provided services and further resulted in improvements in health conditions.^44,45^ Because of improved knowledge, community health practices improved in all sites. This demonstrates the opportunities that lie in capacitating THPs as the first point of contact with many African patients.

The value of having a collective understanding in using concepts and names has been emphasised in this study. Traditional health practitioners stated that there are similarities between concepts used in traditional health practice and Western medicine. This is a significant finding as it breaks some of the barriers and stereotypes that exist regarding the unempirical nature of traditional health practice. The difference may be in the meaning and origin of the concept. However, the symptoms are mostly the same.^4,46^ Indigenous concepts such as *Sefola,* were mentioned by participants, who associated the disease with a sore that does not heal. Steyn and Muller (2000) also reported that THPs in their study referred to *Sefola* as cancer-type sore.^47^ Furthermore, similarities in symptoms between schizophrenia and indigenous diagnosis of *lefu la hloho* [*mental illness*] were reported.^48^ Seemingly, there are great opportunities for translation and interpretation of the terminologies and diagnosis as used by both systems to facilitate mutual understanding. Notably, the treatment approaches and modalities are different as they are influenced by the various philosophies and approaches in the healthcare system. The value of language in naming and interpreting health problems has therefore proven to be the cornerstone for knowledge exchange between THPs and BHPs.

### Strengths and limitations

The findings of our study have the potential to facilitate collaboration between THPs and BHPs by enhancing the referral system between the two systems. The methodology used in this study offers perspectives on how to conduct a needs assessment and offers reference points for THPs and indigenous knowledge holders, which are different from those of BHPs.

## Conclusion

Our study explored the training needs of THPs on biomedical knowledge and skills. Traditional health practitioners who are equipped with complementary knowledge on biomedicine are better positioned to diagnose emergencies, provide first aid interventions and refer patients timely. If THPs are to be capacitated with such knowledge on biomedicine, it would be appropriate to design programmes that are tailor-made to meet the needs of THPs while preserving sacred knowledge systems in traditional health practice. Yet, THPs can apply the knowledge and skills so that their approach to health is holistic while addressing various health challenges brought by patients.

The following recommendations were made based on these findings:

- Designing and provision of courses for THPs geared to assist THPs in recognising emergency situations and to refer timely.
- Evaluate the possibilities of opening medical schools for training of THPs, adopting training models such as those offered by the School of Chinese Medicine in Taiwan^49^ training in first aid for emergencies.
- For research, a large-scale study could yield a greater understanding on the similarities between methods of diagnosis in traditional health practice and biomedical health system.
